# Trends in socio-demographic disparities in COVID-19 vaccine uptake by vaccine dose and time after the introduction of COVID-19 vaccination in Israel: epidemiological and policy analysis study

**DOI:** 10.1186/s13584-026-00758-z

**Published:** 2026-05-04

**Authors:** Hala Manaa, Dani Cohen, Khitam Muhsen

**Affiliations:** Department of Epidemiology and Preventive Medicine, School of Public Health, Gray Faculty of Medical and Health Sciences, Tel Aviv University, 6139001 Ramat Aviv, Tel Aviv, Israel

**Keywords:** COVID-19, Vaccine uptake, Vaccine dose, Israeli population, Social disparities, Ethnic groups, Age groups

## Abstract

**Background:**

Evidence on sociodemographic disparities in COVID-19 booster vaccine uptake remains limited. We examined disparities in COVID-19 vaccine uptake among the Arab, ultraorthodox Jewish, and general Jewish populations in Israel (January 2021-August 2022), focusing on vaccine dose, community characteristics, and policy analysis.

**Methods:**

Publicly available COVID-19 data from 135 cities (≥ 10,000 residents) were analyzed. Cumulative vaccine uptake by dose was assessed by age and population group across 3 periods (period-1: to June 2021, period-2: to December 2021, period-3: to August 2022). Policy analysis was conducted using Walt and Gilson’s Policy Triangle framework, drawing on the literature, government reports, official websites, and media coverage.

**Results:**

Data from 76 predominantly non-ultraorthodox Jewish cities, 10 ultraorthodox Jewish cities, and 49 Arab cities were included. Compared to the general Jewish population, dose 1 uptake was lower in ultraorthodox (incidence rate ratio (IRR) = 0.51, 95% CI 0.26–0.99) and Arab cities: IRR = 0.76, 0.53–1.09), with similar gaps for dose 2. Disparities widened with boosters: for dose 3, uptake was significantly lower in ultraorthodox cities (period-2 IRR = 0.38 [0.20–0.74], period-3: IRR = 0.39 [0.20–0.75]); and Arab cities (period-2 IRR = 0.55 [0.38–0.79], period-3 IRR = 0.56 [0.39–0.81]). For dose 4, gaps were largest in adults aged ≥ 60 years (ultraorthodox: IRR = 0.24 [0.12–0.47]; Arab: IRR = 0.15 [0.10–0.22]). Higher socioeconomic status was consistently associated with uptake, particularly for boosters. The peripherality index was associated with lower dose 4 uptake, suggesting geographic disparities and access barriers. Policy analysis highlighted Israel’s rapid mass vaccination rollout and evidence-based booster adoption, but also declining booster uptake and widening sociodemographic inequities. The primary campaign relied on centralized mass vaccination efforts and intensive public messaging, achieving high coverage, while the booster phase was mainly integrated into existing infrastructure, with more targeted outreach and reduced media emphasis, shifting from infection prevention to severe-disease prevention policy.

**Conclusions:**

Persistent disparities in COVID-19 vaccine uptake widened during the booster phase in Israel. While rapid, centralized rollout achieved high initial coverage, sustaining equitable uptake proved challenging. These findings highlight the need for ongoing, equity-focused strategies, including targeted outreach and culturally tailored interventions throughout all phases of public health emergencies, particularly in the long-term phase, to strengthen existing healthcare infrastructure.

**Supplementary Information:**

The online version contains supplementary material available at 10.1186/s13584-026-00758-z.

## Background

The coronavirus disease 2019 (COVID-19) pandemic has resulted in a profound global impact, with over 777 million confirmed cases and over 7 million deaths as of March 2025 [1].

The COVID-19 vaccines were essential for controlling the pandemic. Israel was among the first countries to introduce COVID-19 vaccination, using the BNT162b2 mRNA vaccine [2, 3] with median first- and second-dose uptake of 89% and 81%, respectively, in individuals aged ≥ 16 years [4]. To address waning immunity and the emergence of the Delta variant of concern, Israel recommended the first booster (third) dose of the COVID-19 vaccine in July 2021 [5, 6]. In response to the surge in COVID-19 during December 2021, attributed to the emergence of the Omicron variant, the Israeli Ministry of Health (MOH) approved the administration of a fourth dose (second booster) of the COVID-19 vaccine to people at high risk, healthcare workers, and individuals aged 60 years or older [7]. This policy was effective in reducing the risk of COVID-19 hospitalizations and severe disease [6–9].

Disparities in COVID-19 vaccine uptake were observed across many countries, with coverage consistently lower among ethnic minority groups [10–14] and among individuals living in deprived areas or with lower income and education levels than those in more affluent groups [14–17]. Evidence on disparities in COVID-19 booster doses remains limited, as does evidence on differences in uptake between doses [10–14]. A survey conducted in 22 countries showed an overall increase in COVID-19 vaccine acceptance between 2020 and 2022; however, in 8 countries, vaccine acceptance decreased [10]. Moreover, 12% of adults who had completed the primary series were hesitant about booster vaccination [10]. This percentage reached 48% in the United States [13]. A study from Norway showed larger gaps in booster dose uptake between underprivileged individuals (lower education and income) and more affluent groups than in the primary vaccination series [12].

In Israel, lower COVID-19 vaccine uptake was reported in the Arab ethnic minority and the religious ultraorthodox Jewish population compared to the general Jewish population [2, 9, 15–17]. Furthermore, COVID-19 uptake increased in cities with higher residential socioeconomic status (SES) [9, 16, 17], with limited evidence suggesting increased social disparities in booster dose uptake [18]. Early in the COVID-19 vaccination campaign, a coordinated nationwide effort was launched in Israel to ensure vaccine accessibility, including the establishment of widespread vaccination centers with extended opening hours, targeted public messaging, extensive and multi-faceted targeting of outreach to the Arab and ultraorthodox populations, including in the framework of designated COVID-19 task forces in these population groups, and advertising, and implementing the "Green Pass"^1^ policy to encourage vaccination [2, 19]. Gradually, these efforts faded, and with the easing of COVID-19 restrictions [20], the sense of urgency around vaccination has declined. Hence, we examined trends in gaps of COVID-19 vaccine uptake between the Arab, ultraorthodox Jewish, and general Jewish populations in Israel between January 2021 and August 2022, across vaccine doses and age groups, while exploring the impact of community-level sociodemographic characteristics. Additionally, we conducted a policy analysis of COVID-19 vaccination in Israel, comparing primary versus booster dose policies and assessing their policy implications for vaccine equity.

## Methods

### Study design and population

We analyzed publicly available national COVID-19 data from the Israeli MOH. In 2021, Israel’s population of approximately 9.5 million people included 74% Jews, 21% Arabs, and 5% other ethnic groups [21]; about 13% of the population is estimated as ultraorthodox Jewish [22]. Arab and ultraorthodox Jewish communities are generally younger, have higher fertility rates, lower SES compared to the general Jewish population [22, 23], and often live in separate localities.

By April 2025, approximately 4.9 million people confirmed severe acute respiratory syndrome coronavirus 2 (SARS-CoV-2) infections, and 13,171 COVID-19-related deaths were reported in Israel [4]. The uptake rates of SARS-CoV-2 PCR tests were initially lower in Arab towns [9, 24], while infection and mortality rates were higher among lower-SES groups [9] and predominantly Arab communities than in cities with predominantly Jewish residents [9], and declined in higher-SES cities [25].

COVID-19 vaccination became available to all individuals aged ≥ 16 years in Israel in January 2021. In July 2021, vaccination with the first booster (third dose) was introduced, and in December 2021, the second booster (fourth dose) was recommended for individuals aged ≥ 60 years, high-risk groups, healthcare workers, and subsequently became available to younger individuals.

### Data sources

Aggregate COVID-19 data were obtained from the Israeli MOH in 281 towns/cities. Of those, 135 had ≥ 10,000 residents and were included in the study. Data included the number of confirmed SARS-CoV-2 infections (primarily by rt-PCR), COVID-19 testing, and vaccine uptake (first through fourth doses) in each town, overall, and by age group [26, 27]. To examine trends in vaccination gaps, data were categorized into three periods: Period 1: March 2020- June 2021 (wild-type SARS-CoV-2 and Alpha variant of concern, a 2-dose schedule); Period 2: July 2021-December 2021 (Delta variant wave, introduction dose 3 (first booster)); Period 3: January-August 2022 (Omicron wave, introduction dose 4 (second booster)).

Sociodemographic data were obtained from the Israel Central Bureau of Statistics (ICBS).

### The outcome variable

The outcome variables were the cumulative rate (per 1000 residents) of the first through fourth COVID-19 vaccine doses in each town, analyzed by study period and age group (20–29, 30–59, and ≥ 60 years).

### The independent variables

The independent variables included town-level sociodemographic characteristics: predominant population group (general population, Jewish, ultraorthodox Jewish, or Arabs, SES rank, age groups (20–29, 30–59, and ≥ 60 years), and peripherality index.

The Arab cities included 100% Arab residents. The Jewish cities included > 95% Jewish residents according to ICBS, except for 7 cities (Haifa, Malalot-Tarshiha, Ramla, Lod, Acre, and Jerusalem) that included 12%-38% Arab residents, which we classified as cities with predominantly Jewish residents [28]. The towns with predominantly Jewish residents were further classified into general Jewish population and mainly ultraorthodox cities if the estimated ultraorthodox Jewish population was 48%-99% (median = 80% of the Jewish population in the city) [23, 28, 29], which included Beit Shemesh, Bnei Brak, Beitar Illit, El’ad, Givat Zeev, Jerusalem, Modi’in Illit, Netivot, Rekhasim, and Zefad [28]. SES rank was defined using the ICBS classification, on an ordinal scale from 1 to 10, with higher values indicating higher SES [24, 24]. The peripherality index of the town of residence was determined using the ICBS classification, on an ordinal scale from 1 to 10, where lower values indicate higher peripherality [30].

Additional variables included population density, average salary (employees and self-employed), cumulative SARS-CoV-2 infection rates, and COVID-19 testing rates (per 10,000 residents) to describe the study population and contextualize vaccine uptake in the broader COVID-19 epidemiology and health behaviors.

### Statistical analysis

Differences in socioeconomic factors across population groups were assessed using the Kruskal–Wallis test. The cumulative confirmed SARS-CoV-2 infections and diagnostic testing rate were calculated per 10,000 residents, and vaccine uptake rates (per 1,000 residents) were calculated overall, by age group, and by study period. Correlations among independent variables using Spearman’s rank correlation coefficient. Associations between community-level factors and vaccine uptake were analyzed using generalized linear models with the negative binomial distribution, reporting incidence (uptake) rate ratios (IRRs) and 95% confidence intervals (CIs). Highly correlated variables were analyzed separately to avoid multicollinearity. Analyses were stratified by vaccine dose, age group, and period and conducted using IBM SPSS (IBM, Armonk, New York, NY, USA) software (version 29).

### Policy analysis

We conducted a policy analysis of Israel’s COVID-19 vaccination strategy and other public health measures, including descriptive and comparative analyses and equity-focused components. The policy analysis was guided by Walt and Gilson’s Policy Triangle framework (context, content, process, and actors) [31].

We reviewed scientific literature, governmental and non-governmental reports, MOH publications, and media sources to examine vaccination rollout strategy, infrastructure, and outreach efforts. We assessed how policies addressed or potentially exacerbated sociodemographic inequalities in vaccine uptake and compared implementation across primary series/first campaign and booster dose campaign to identify equitable vaccination.

## Results

### Description of the study population

We included 135 towns (≥ 10,000 residents); 49 (36.3%) predominantly Arab and 86 (63.7%) predominantly Jewish, of which 10 were predominantly ultraorthodox Jewish. Population size ranged from 10,394 to 981,711 (median = 28,169). Ultraorthodox Jewish towns had the highest density (p < 0.001). Median salary and residential SES rank were higher in the general Jewish cities than in the ultraorthodox Jewish and Arab towns (p < 0.001). The peripherality index was lower in the Arab towns than in Jewish cities (Table 1).

**Table 1:** Sociodemographic characteristics of study cities by population group

Variable	Overall	General jewish population	Ultraorthodox jewish population	Arab population	P value
Number of towns	135	76	10	49	
Total population	28,169 (40,474)	42,167 (53,516)	56,751 (136,814)	18,190 (11,334)	Kruskal–Wallis H = 38.176, p < 0.001)
Min–max	10,394–981,711	10,993–474,530	14,198–981,711	10,394–79,064	
Population density, residents/ km^2^	2,713 (2606)	3,376 (3,493)	4,288 (11,334)	2,260 (1097)	Kruskal–Wallis H = 18.738, p < 0.001
Min–max	166–29,714	166–19,098	1,270.2–29,714	744–9,396	
Average monthly salary -Employees, NIS	7,920 (4,090)	9,955 (3,814)	6,537 (1,756)	6,425(826)	Kruskal–Wallis H = 90.930, p < 0.001
Min–max	5,157–15,412	6,912–15,412	5,157–8,953	5,395–8,151	
Average monthly salary- self-employed, NIS	9,181 (2,669)	10,430 (2,937)	7,848 (2,113)	8,579 (1,290)	Kruskal–Wallis H = 46.368, p < 0.001
Min–max	5,901–14,639	7,483–14,639	5,901–9,683	5,945–12,375	
Residential SES rank	5 (4)	7 (3)	2 (1)	3 (2)	Kruskal–Wallis H = 86.917, p < 0.001
Min–max	1–9	3–9	1–5	1–5	
Peripherality index	5 (3)	6 (3)	6 (3)	4 (2)	Kruskal–Wallis H = 30.098, p < 0.001
Min–max	1–10	1–10	2–10	1–7	

The data presented are medians and interquartile range (IQR). Data on population density were missing for 6 towns. Min-minimum, max-maximum, km^2^-square kilometer, NIS-new Israeli shekels, SES-socioeconomic status.

Average monthly employee salary was strongly correlated with residential SES rank (r = 0.959) and self-employed income (r = 0.729), and SES rank also correlated with self-employed income (r = 0.722) (Additional file 1). Therefore, we included population group, residential SES rank, and peripherality index as the independent variables.

### Incidence of SARS-CoV-2 infection and COVID-19 testing uptake

Between March 2020 and June 2021, the median cumulative SARS-CoV-2 infection rate was higher in the ultraorthodox Jewish communities compared to the general Jewish population cities; adjusted IRR = 2.79 (95% CI 1.44–5.41), and higher in Arab cities, although the association was not statistically significant: IRR = 1.40 (0.97–2.00). Results were similar in period 2. In period 3, cumulative infection rates were highest in the general Jewish cities, followed by the ultraorthodox Jewish and Arab populations, but the differences were not statistically significant. Testing uptake was higher in the general Jewish cities. SES was inversely associated with infection rates in periods 1 and 2, but positively associated in period 3 (Additional file 2 and Additional file 3).

### Associations between community-level sociodemographic factors and COVID-19 vaccine uptake, by dose and period (all ages combined)

The results of the bivariate analyses are presented in Additional file 4.

**Fig. 1:**
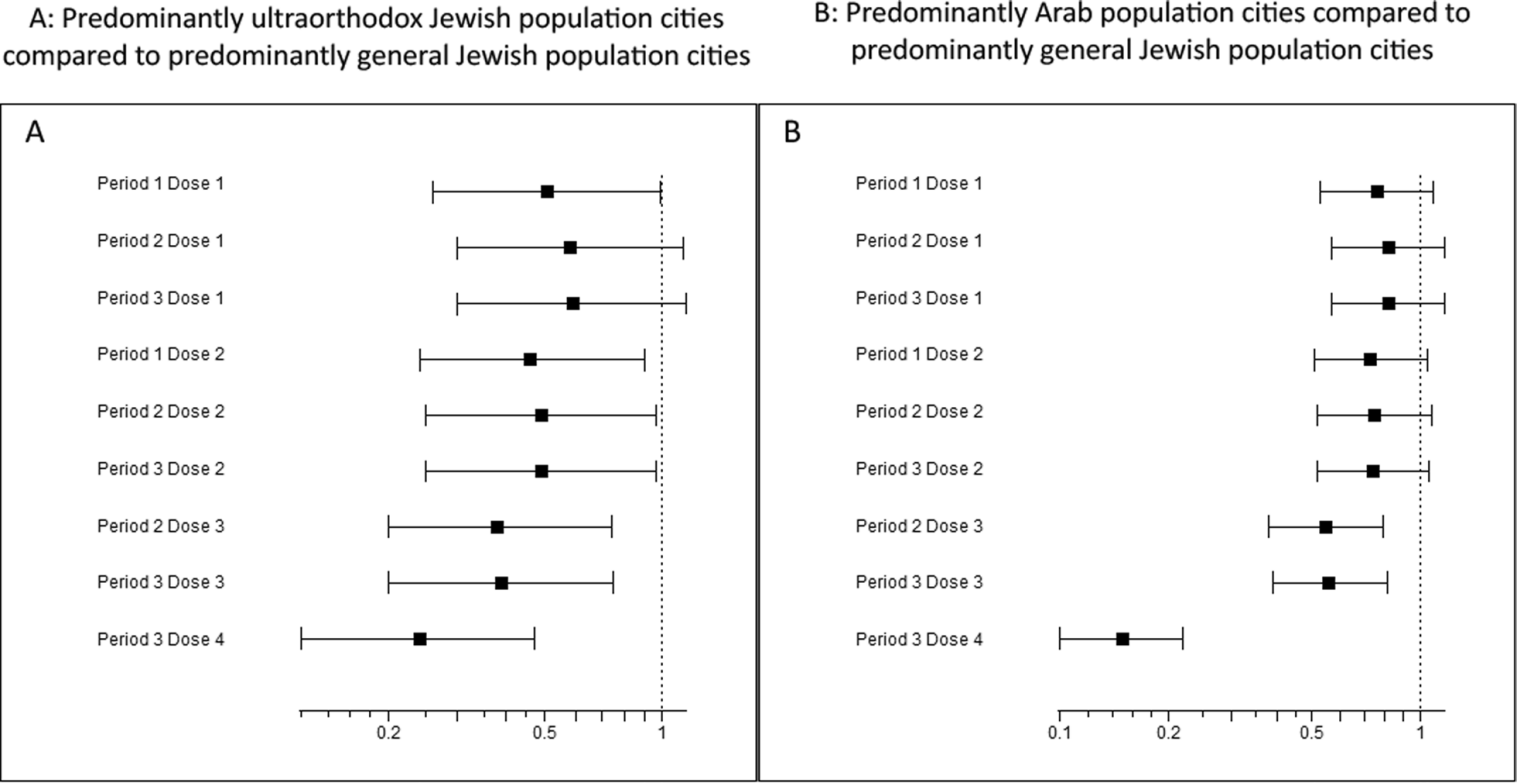
Adjusted incidence rate ratio and 95% confidence intervals for the COVID-19 vaccine uptake by period and dose. Panel (**A**) compares predominantly ultraorthodox Jewish population cities with predominantly general Jewish population cities. Panel (**B**) compares predominantly Arab-population cities with predominantly Jewish-population cities

In adjusted all-ages analyses, ultraorthodox cities had a significantly lower uptake of dose 1 in period 1 than the general Jewish population cities (IRR = 0.51, 95% CI 0.26–0.99) (p = 0.048), with no significant differences in later periods. Arab cities showed a lower uptake across periods, but the differences were not statistically significant. For dose 2, uptake was significantly lower in ultraorthodox cities in period 1, IRR = 0.46 (0.24–0.90) (p = 0.024), and marginally lower in Arab cities, IRR = 0.73 (0.51–1.05), p = 0.091, with similar results in later periods. Dose 3 uptake (from period 2) was significantly lower in ultraorthodox (IRR = 0.38, 0.20–0.74) (p = 0.005) and Arab cities (IRR = 0.55, 0.38–0.79), (p = 0.001) compared to general Jewish cities, with comparable findings in period 3. In period 3, dose 4 uptake was markedly lower in ultraorthodox (IRR = 0.24, 0.12–0.47) p < 0.001 and Arab cities (0.15, 0.10–0.22), p <0.001 compared to the general Jewish population cities (Table 2, Fig. 1).

**Table 2:** Adjusted associations of residential sociodemographic factors with all-ages combined COVID-19 vaccine uptake by dose and period

	Uptake of COVID-19 vaccine–dose 1 all ages		Uptake of COVID-19 vaccine–dose 2 all ages		Uptake of COVID-19 vaccine–dose 3 all ages		Uptake of COVID-19 vaccine–dose 4 all ages	
	IRR (95% CI)	P value	IRR (95% CI)	P value	IRR (95% CI)	P value	IRR (95% CI)	P value
**Period 1**								
Population group		**0.081**		**0.037**	NA		NA	
General jewish population towns	Reference		Reference		NA		NA	
Ultraorthodox towns	**0.51 (0.26–0.99)**	**0.048**	**0.46 (0.24–0.90)**	**0.024**	NA		NA	
Arab towns	0.76 (0.53–1.09)	0.147	0.73 (0.51–1.05)	0.091	NA		NA	
SES rank	**1.09 (1.01–1.17)**	**0.025**	**1.10 (1.02–1.19)**	**0.012**	NA		NA	
Peripherality index	1.03 (0.95–1.11)	0.421	1.03 (0.95–1.12)	0.374	NA		NA	
**Period 2**								
Population group		0.216		**0.062**		** < 0.001**		
General jewish population towns	Reference		Reference		Reference		NA	
Ultraorthodox towns	0.58 (0.30–1.13)	0.113	**0.49 (0.25–0.96)**	**0.038**	**0.38 (0.20–0.74)**	**0.005**	NA	
Arab towns	0.82 (0.57–1.17)	0.287	0.75 (0.52–1.08)	0.124	**0.55 (0.38–0.79)**	**0.001**	NA	
SES rank	**1.07 (0.99–1.16)**	**0.062**	**1.09 (1.01–1.18)**	**0.017**	**1.17 (1.08–1.27)**	** < 0.001**	NA	
Peripherality index	1.02 (0.94–1.11)	0.510	1.03 (0.95–1.12)	0.389	1.06 (0.98–1.15)	0.106	NA	
**Period 3**								
Population group		0.228		**0.057**		** < 0.001**		** < 0.001**
General jewish population towns	Reference		Reference		Reference		Reference	
Ultraorthodox towns	0.59 (0.30–1.15)	0.124	**0.49 (0.25–0.96)**	**0.037**	**0.39 (0.20–0.75)**	**0.005**	**0.24 (0.12–0.47)**	** < 0.001**
Arab towns	0.82 (0.57–1.17)	0.281	0.74 (0.52–1.06)	0.109	**0.56 (0.39–0.81)**	**0.002**	**0.15 (0.10–0.22)**	** < 0.001**
SES rank	**1.07 (0.99–1.15)**	**0.062**	**1.10 (1.02–1.18)**	**0.013**	**1.17 (1.08–1.26)**	** < 0.001**	**1.45 (1.33–1.58)**	** < 0.001**
Peripherality index	1.02 (0.94–1.11)	0.503	1.03 (0.95–1.12)	0.363	1.06 (0.98–1.15)	0.115	**1.17 (1.09–1.27)**	** < 0.001**

CI: confidence interval; COVID-19: coronavirus disease 2019; IRR: Incident rate ratio; SES: socioeconomic status. Results from multivariable models. The peripherality index and SES were analyzed in separate models due to the high correlation. Bold values: P value <0.1

**Fig. 2:**
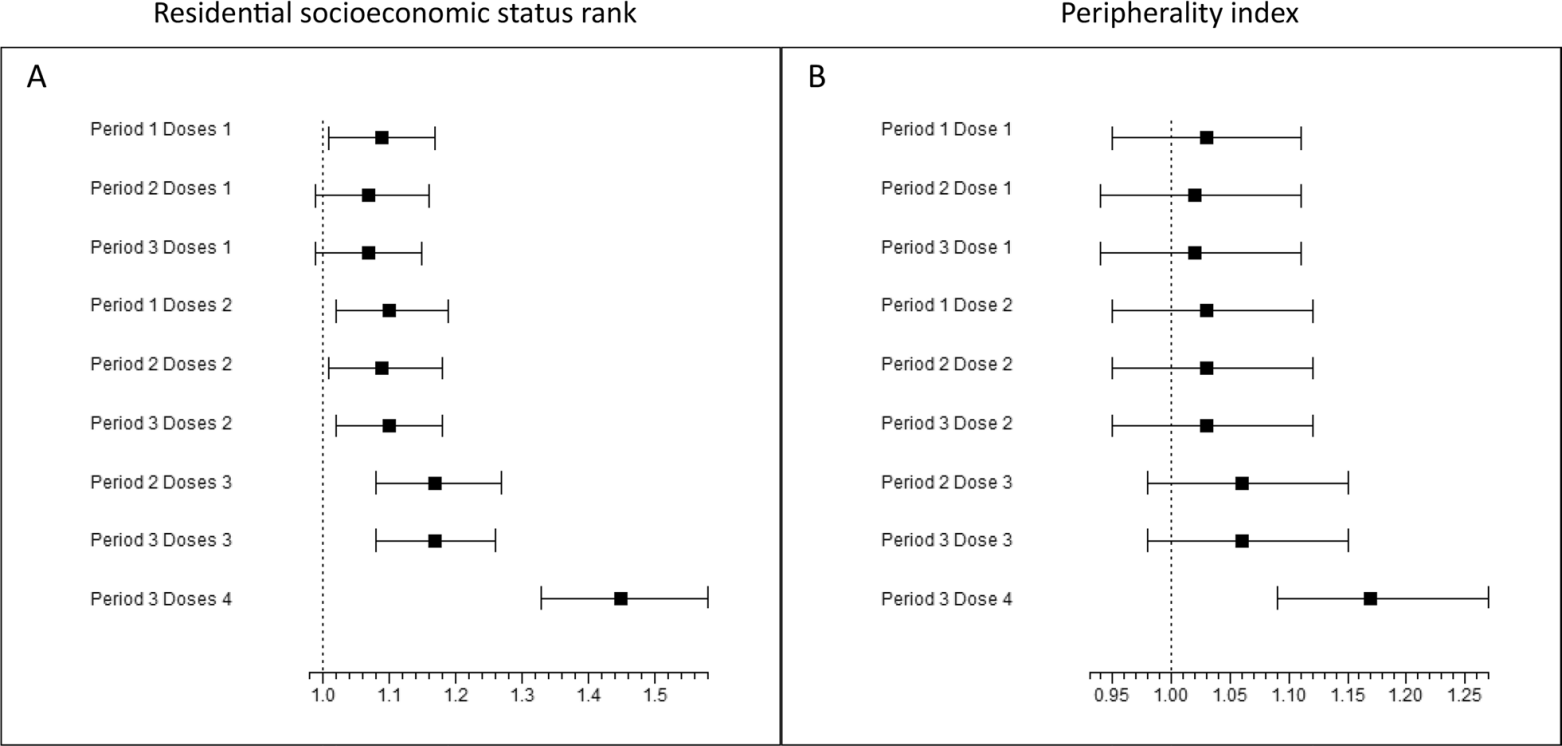
Adjusted incidence rate ratio and 95% confidence intervals for the COVID-19 vaccine uptake by period and dose Panel (**A**) represents the association between residential socioeconomic status rank and vaccine uptake. Panel (**B**) represents the association between the residential peripherality index and vaccine uptake

Higher residential SES rank was positively associated with COVID-19 vaccine uptake for all doses and periods (except dose 1 in periods 2–3), with the strongest association observed for dose 4: IRR = 1.45 (1.33–1.58), p <0.001. The peripherality index was not associated with the uptake of doses 1–3, but was positively associated with dose 4 uptake, IRR = 1.17 (1.09–1.27), p <0.001 (Table 2, Fig. 2).

### Age-stratified associations between community-level sociodemographic factors and COVID-19 vaccine uptake by dose and period

The age-stratified analyses showed no significant associations between population groups and doses 1–2 uptake, but significant associations were found for doses 3–4, with weaker differences among adults ≥ 60 years. Residential SES was not associated with dose 1 uptake; however, it was significantly (p = 0.044) positively associated with dose 2 uptake among the 20–29-year-olds in period 1, and was positively associated with doses 3–4 uptake across age groups and periods (p <0.05). The peripherality index was not associated with doses 1–3 uptake, but positively associated with dose 4 uptake in all age groups (p <0.001) (Tables 3, 4, 5 and 6).

**Table 3:** Adjusted associations of residential sociodemographic factors with overall and age-specific COVID-19 vaccine dose 1 uptake by period

	Uptake of COVID-19 vaccine-dose 1 age 20–29 years		Uptake of COVID-19 vaccine-dose 1 age 30–59 years		Uptake of COVID-19 vaccine-dose 1 age ≥ 60 years	
	IRR (95% CI)	P value	IRR (95% CI)	P value	IRR (95% CI)	P value
**Period 1**						
Population group		0.346		0.437		0.505
General Jewish population towns	Reference		Reference		Reference	
Ultraorthodox towns	0.64 (0.33–1.24)	0.190	0.70 (0.36–1.35)	0.290	0.86 (0.44–1.66)	0.660
Arab towns	0.84 (0.59–1.21)	0.366	0.84 (0.58–1.20)	0.347	0.81 (0.56–1.16)	0.249
SES rank	1.06 (0.98–1.14)	0.106	1.05 (0.97–1.13)	0.173	1.04 (0.97–1.12)	0.245
Peripherality index	1.02 (0.94–1.10)	0.572	1.01 (0.94–1.10)	0.655	1.02 (0.94–1.11)	0.526
**Period 2**						
Population group		0.605		0.655		0.590
General Jewish population towns	Reference		Reference		Reference	
Ultraorthodox towns	0.73 (0.37–1.41)	0.356	0.77 (0.40–1.49)	0.449	0.88 (0.45–1.71)	0.725
Arab towns	0.89 (0.62–1.28)	0.562	0.88 (0.61–1.26)	0.503	0.83 (0.57–1.18)	0.309
SES rank	1.04 (0.97–1.12)	0.245	1.03 (0.96–1.12)	0.312	1.03 (0.96–1.11)	0.312
Peripherality index	1.01 (0.93–1.10)	0.691	1.01 (0.93–1.09)	0.748	1.02 (0.94–1.10)	0.575
**Period 3**						
Population group		0.623		0.667		0.594
General Jewish population towns	Reference		Reference		Reference	
Ultraorthodox towns	0.73 (0.38–1.42)	0.367	0.77 (0.40–1.50)	0.456	0.89 (0.46–1.72)	0.731
Arab towns	0.90 (0.63–1.29)	0.581	0.88 (0.62–1.27)	0.514	0.83 (0.58–1.19)	0.312
SES rank	1.04 (0.96–1.12)	0.260	1.03 (0.96–1.11)	0.323	1.03 (0.96–1.11)	0.318
Peripherality index	1.01 (0.93–1.10)	0.702	1.01 (0.93–1.09)	0.757	1.02 (0.94–1.10	0.579

CI: confidence interval, COVID-19: coronavirus disease 2019, IRR: Incident rate ratio, SES: socioeconomic status. The peripherality index and SES were analyzed in separate models due to the high correlation.

**Table 4:** Adjusted associations of residential sociodemographic factors with overall and age-specific COVID-19 vaccine dose 2 uptake by period

	Uptake of COVID-19 vaccine-dose 2 age 20–29 years		Uptake of COVID-19 vaccine-dose 2 age 30–59 years		Uptake of COVID-19 vaccine-dose 2 age ≥ 60 years	
	IRR (95% CI)	P value	IRR (95% CI)	P value	IRR (95% CI)	P value
**Period 1**						
Population group		0.182		0.255		0.322
General Jewish population towns	Reference		Reference		Reference	
Ultraorthodox towns	0.58 (0.30–1.12)	0.105	0.63 (0.32–1.22)	0.173	0.80 (0.41–1.55)	0.517
Arab towns	0.80 (0.55–1.14)	0.225	0.80 (0.56–1.14)	0.229	0.76 (0.53–1.09)	0.143
SES rank	**1.08 (1.00–1.16)**	**0.044**	1.06 (0.99–1.15)	0.091	1.05 (0.98–1.14)	0.138
Peripherality index	1.03 (0.95–1.11)	0.475	1.02 (0.94–1.10)	0.557	1.03 (0.95–1.11)	0.453
**Period 2**						
Population group		0.279		0.336		0.370
General Jewish population towns	Reference		Reference		Reference	
Ultraorthodox towns	0.61 (0.31–1.18)	0.142	0.66 (0.34–1.28)	0.225	0.83 (0.43–1.62)	0.600
Arab towns	0.84 (0.58–1.20)	0.350	0.82 (0.57–1.17)	0.279	0.77 (0.54–1.11)	0.165
SES rank	1.06 (0.99–1.15)	0.088	1.06 (0.98–1.14)	0.130	1.05 (0.97–1.13)	0.173
Peripherality index	1.02 (0.94–1.10)	0.562	1.02 (0.94–1.10)	0.611	1.03 (0.95–1.11)	0.467
**Period 3**						
Population group		0.295		0.354		0.395
General Jewish population towns	Reference		Reference		Reference	
Ultraorthodox towns	0.61 (0.31–1.19)	0.151	0.67 (0.34–1.29)	0.237	0.84 (0.43–1.63)	0.617
Arab towns	0.84 (0.59–1.21)	0.364	0.82 (0.57–1.18)	0.291	0.78 (0.54–1.11)	0.178
SES rank	1.06 (0.98–1.14)	0.095	1.05 (0.98–1.14)	0.139	1.05 (0.97–1.13)	0.189
Peripherality index	1.02 (0.94–1.10)	0.570	1.02 (0.94–1.10)	0.619	1.02 (0.95–1.11)	0.484

CI: confidence interval, COVID-19: coronavirus disease 2019, IRR: Incident rate ratio, SES: socioeconomic status. The peripherality index and SES were analyzed in separate models due to the high correlation. Bold value: P value <0.05

**Table 5:** Adjusted associations of residential sociodemographic factors with overall and age-specific COVID-19 vaccine dose 3 uptake by period

	Uptake of COVID-19 vaccine-dose 3 age 20–29 years		Uptake of COVID-19 vaccine-dose 3 age 30–59 years		Uptake of COVID-19 vaccine-dose 3 age ≥ 60 years	
	IRR (95% CI)	P value	IRR (95% CI)	P value	IRR (95% CI)	P value
**Period 2**						
Population group		**0.007**		**0.016**		**0.044**
General Jewish population towns	Reference		Reference		Reference	
Ultraorthodox towns	**0.44 (0.23–0.86)**	**0.017**	**0.54 (0.28–1.04)**	**0.068**	0.76 (0.39–1.48)	0.434
Arab towns	**0.62 (0.43–0.90)**	**0.011**	**0.62 (0.43–0.89)**	**0.010**	**0.63 (0.44–0.90)**	**0.013**
SES rank	**1.15 (1.06–1.24)**	** < 0.001**	**1.12 (1.04–1.21)**	**0.002**	**1.09 (1.01–1.18)**	**0.016**
Peripherality index	1.06 (0.97–1.14)	0.155	1.05 (0.97–1.13)	0.211	1.05 (0.97–1.13)	0.222
**Period 3**						
Population group		**0.014**		**0.023**		**0.049**
General Jewish population towns	Reference		Reference		Reference	
Ultraorthodox towns	**0.45 (0.23–0.88)**	**0.020**	**0.54 (0.28–1.05)**	**0.072**	0.77 (0.40–1.50)	0.453
Arab towns	**0.66 (0.46–0.95)**	**0.025**	**0.64 (0.44–0.92)**	**0.016**	**0.63 (0.44–0.91)**	**0.014**
SES rank	**1.13 (1.05–1.22)**	** < 0.001**	**1.12 (1.04–1.21)**	**0.003**	**1.09 (1.01–1.18)**	**0.019**
Peripherality index	1.05 (0.97–1.14)	0.199	1.04 (0.96–1.13)	0.239	1.05 (0.97–1.13)	0.229

CI: confidence interval, COVID-19: coronavirus disease 2019, IRR: Incident rate ratio, SES: socioeconomic status. The peripherality index and SES were analyzed in separate models due to the high correlation. Bold value: P value <0.05

**Table 6:** Adjusted associations of residential sociodemographic factors with overall and age-specific COVID-19 vaccine dose 4 uptake- period 3

	Uptake of COVID-19 vaccine – dose 4- age 20–29 years		Uptake of COVID-19 vaccine – dose 4- age 30–59 years		Uptake of COVID-19 vaccine – dose 4–age ≥ 60 years	
	IRR (95% CI)	P value	IRR (95% CI)	P value	IRR (95% CI)	P value
**Period 3**						
Population group		< 0.001		< 0.001		< 0.001
General Jewish population towns	Reference		Reference		Reference	
Ultraorthodox towns	0.28 (0.14–0.55)	< 0.001	0.36 (0.18–0.69)	0.002	0.56 (0.29–1.08)	0.087
Arab towns	0.35 (0.24–0.51)	< 0.001	0.27 (0.19–0.39)	< 0.001	0.26 (0.18–0.37)	< 0.001
SES rank	1.27 (1.18–1.37)	< 0.001	1.30 (1.20–1.40)	< 0.001	1.27 (1.17–1.36)	< 0.001
Peripherality index	1.15 (1.06–1.25)	< 0.001	1.14 (1.05–1.23)	< 0.001	1.13 (1.05–1.22)	0.001

CI: confidence interval, COVID-19: coronavirus disease 2019, IRR: Incident rate ratio, SES: socioeconomic status. The peripherality index and SES were analyzed in separate models due to the high correlation

### Policy analysis

**Fig. 3:**
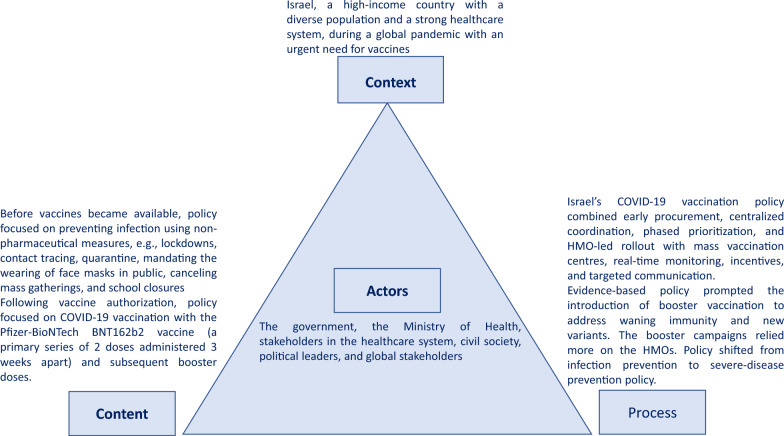
Israel COVID-19 vaccination policy analysis using Walt and Gilson’s policy triangle framework [31]

Using the health policy triangle, Israel’s COVID-19 vaccination campaign can be viewed as follows (Fig. 3):

### Context

Israel’s universal national health insurance system provides comprehensive care [32] through 4 health maintenance organizations (HMOs) under MOH regulations, supported by a robust electronic health record infrastructure [33]. Despite these strengths, disparities in access to health services persist between peripheral regions and the center of Israel [32]. Routine childhood vaccines are administered via maternal and child health clinics, while adult vaccines are delivered through HMO primary care clinics. Coverage of routine childhood vaccines is lower among the ultraorthodox Jewish population and higher among the Arab population than in the general Jewish population [34, 35], whereas adult vaccine uptake is lower in both Arab and ultraorthodox Jewish populations relative to the general Jewish population [36].

Early political commitment and dedicated funding enabled the rapid and early procurement of COVID-19 vaccines [2, 3, 37].

### Policy content

Before COVID-19 vaccines became available, the main policy focused on preventing the spread of infection and interrupting transmission chains [9, 20, 38] by implementing lockdowns, testing expansion, contact tracing, quarantining SARS-CoV-2-infected individuals, mandating the wearing of face masks in public, and banning mass gatherings and school closures [9, 38]. During this period, Israel established a central electronic surveillance system for COVID-19 [9, 38].

The COVID-19 vaccination policy aimed to prevent severe disease and enable economic reopening. It relied on Pfizer-BioNTech BNT162b2 vaccine (a primary series of 2 doses, administered 21 days apart), given via mass and community vaccination centers, mobile clinics, implementing the “Green Pass” incentive program, extensive advertising campaigns, and booster vaccination [2, 3, 19, 20], introduced later following evidence waning immunity [5, 19, 20].

### Actors

The COVID-19 pandemic management relied on inter-ministries collaboration led by the Prime Minister and the Corona Ministers Committee, alongside MOH professional leadership led by the Director General and Public Health Services officers, who collaborated with additional actors in the health care system and across ministries [38].

The MOH directed the national vaccination strategy, collaborating with the Epidemic Management Team (EMT), the National Immunization Technical Advisory Group (NITAG), and HMOs [2, 3, 20, 37, 38]. Additional actors included political leaders, the military, the media, municipalities, and community leaders among minority populations [20, 37]. MOH established a special task force (Magen Israel) to manage the pandemic across the entire population, and specific task forces for sub-population groups at high risk for disease, including the Arab population and the ultraorthodox Jewish population task force, which comprised health professionals, communication experts, civil society organizations, and local authorities. The MOH also established the Senior Shield task force to protect residents in long-term care facilities [6, 8]. International actors included the WHO, the FDA, and Pfizer, Israel’s main vaccine provider [20, 38].

### Process

Rapid vaccine deployment was central to Israel’s pandemic response. Early agreements with vaccine manufacturers ensured sufficient supply upon authorization. The agreement with Pfizer secured sufficient supply for all individuals aged ≥ 16 years, supported by major financial investment [37] and cold-chain preparations [2, 3].

The EMT and NITAG established a clear, phased prioritization plan, vaccinating healthcare workers, older adults, and individuals with underlying conditions first, followed by younger age groups in a graded manner [2, 3].

Vaccination was primarily delivered by HMOs through mass vaccination centers and clinics with extended operating hours, accounting for approximately 87% of doses by March 2022 [2, 37]. HMOs received financial incentives to meet vaccination coverage targets [37]. Hospitals vaccinated healthcare workers early, while the Senior Shield task force vaccinated residents and healthcare workers in long-term care facilities [6, 8, 39].

The MOH managed the campaign through the Center for Control, Knowledge, Logistics, and Operations, providing real-time evidence on vaccination progress, impact, and effectiveness [2, 37]. Mass media and multilingual communication campaigns countered misinformation and raised public awareness about vaccine safety and efficacy [37, 40], coupled with the Green Pass policy [2, 19, 20, 41], promoted vaccine uptake.

The success of the vaccination program required monitoring vaccine uptake and effectiveness in real-world settings, as well as assessing the incidence of post-vaccination adverse events. Such studies were undertaken by the MOH and HMOs, in collaboration with academic partners [6, 8, 42–44], owing to the strong infrastructure of electronic health records and surveillance systems.

### Outcomes and evidence

Israel rapidly achieved high vaccine uptake, ranking among the top countries internationally [2, 3] and generated influential real-world evidence demonstrating high vaccine effectiveness against SARS-CoV-2 infection, severe disease, hospitalization, and mortality [19, 43], supporting reopening of the economy and informing global policy [3]. Vaccine safety monitoring and the early detection of rare post-vaccination adverse events, such as myocarditis, informed real-time policy decisions [20, 37, 44]. Nonetheless, disparities in vaccine uptake were observed. Ultraorthodox Jewish and Arab populations, as well as lower socioeconomic status communities, had lower vaccination rates compared to the general Jewish population [9, 15, 16, 40]. Geographic disparities between peripheral and central regions also emerged during the booster campaign, as we demonstrated herein. For the primary series, these gaps were particularly large at the beginning of the campaign and then narrowed over time [40].

The MOH coordinated culturally tailored interventions to reduce these gaps, collaborating with HMOs, municipalities, and community leaders. Measures included increasing vaccination sites in underserved areas, establishing mobile clinics, extending operating hours, and launching communication campaigns that addressed specific community concerns.

### Booster vaccination policy

By mid-2021, Israel introduced a booster vaccination using BNT162b2 [20] in response to waning immunity and the emergence of the Delta variant, initially targeting older adults and healthcare workers and later expanded to all age groups [21, 22]. In December 2021, Israel became the first country to approve a second booster during the Omicron wave [7, 8].

### Comparative analysis of the primary and booster vaccination campaigns

The first vaccination campaign (December 2020-June 2021) involved rapid, centralized, emergency deployment through HMOs, mainly community clinics, mass vaccination centers [37, 45], hospital-based vaccination sites [37], and the Senior Shield task force [6, 8]. During the first campaign, Clalit Health Services established 400 vaccination centers nationwide, and Maccabi Healthcare Services began with 24 designated vaccination centers, aiming to expand to 80. Similar preparedness operations were implemented by the Meuhedet and Leumit HMOs [45]. The large-scale implementation and extensive mobilization of health resources and infrastructure enabled achieving high vaccination coverage during this emergency phase [3, 40]. This period was characterized by the “prevention of infection policy” [26], under which a lockdown was imposed until the end of February 2021, along with other non-pharmaceutical public measures [9].

The booster campaign (July 2021–2022) was launched as an evidence-based policy to address waning immunity and the emergence of new variants [20], with vaccination integrated into the routine HMO infrastructure [46], targeted outreach to high-risk populations, and limited reliance on mass vaccination centers that were opened as demand increased [47].

Campaign messaging differed between phases. The initial campaign framed vaccination as an urgent national mission, emphasizing collective responsibility (“Give a shoulder” campaign) and extensive media coverage [48], as well as visible leadership endorsement [40]. During the booster phase, media coverage declined [49], and messaging shifted to evidence-based policy risk-focused recommendations, highlighting waning immunity and new variants emergence [20]. Both positive and fear-messaging delivered by the healthcare system and the mass news media increased around the launch of vaccination campaigns [49]. The policy focus also evolved from preventing infection to preventing severe illness, as most of the population was vaccinated and the treatments became available [20].

### Strengths, challenges, and policymaking

Israel’s vaccination policy was characterized by rapid rollout, centralized coordination, high uptake, data-driven decision-making, and transparent communication. However, booster uptake declined relative to the primary series [20, 37], and disparities widened across population groups, socioeconomic status, and regions.

Israel’s COVID-19 vaccination policy provides key lessons for the design and implementation of equitable vaccination strategies in future public health emergencies. Preparedness plans should proactively address the sociodemographic and cultural diversity of the population, anticipating potential gaps in vaccine access and acceptance across population groups, socioeconomic strata, and geographic regions.

Operational strategies should include sustained, dedicated resources for culturally tailored ongoing interventions, and engagement with community leaders (e.g., religious leaders, municipal officials), local influencers, and trusted communication channels to convey accurate information and foster public trust. Expanding mobile vaccination units and mass vaccination centers beyond the initial vaccination campaigns, extending clinic hours, and establishing additional vaccination sites in underserved areas can further reduce barriers to access, especially in the longer-term phases of a health emergency, when invested efforts and resources often fade.

Financial incentives, such as “support tests”^2^ for healthcare providers, could be targeted to increase booster uptake among underserved subpopulations, including older adults and high-risk groups. More broadly, financial incentives should be considered to reduce vaccine inequities across sub-populations and socioeconomic groups.

Ultimately, rapid data collection, real-time monitoring, and transparent communication are crucial for informing adaptive policy decisions, identifying emerging disparities, and fostering public confidence in vaccines. Lessons from Israel underscore that even highly successful vaccination campaigns must anticipate and actively address inequities or even widening gaps, to ensure inclusive protection for all population segments.

The policy shift, from preventing infection to preventing severe illness, in the first vaccination and booster vaccination campaigns is natural and justified, underscoring the need to integrate longer-term COVID-19 vaccination into the existing healthcare infrastructure. In future similar emergencies, it is recommended that such a transition to the longer-term phase be accompanied by sustained, intensified resources, such as manpower, mobile and mass vaccination centers in peripheral geographic regions, and in the Arab and ultraorthodox Jewish populations, to minimize access barriers. The intensity of media coverage should also be increased in the “longer-term” phase, particularly among these populations, to ensure the correct messaging reaches them, increases trust, health literacy, and vaccine uptake.

## Discussion

We examined disparities in COVID-19 vaccine uptake in Israel by population group, age, dose, and period, and assessed the roles of residential SES and peripherality, complemented by a policy analysis.

The main findings of our study are persistent gaps in COVID-19 vaccine uptake between sub-population groups and by residential SES, which widened as booster vaccination was introduced. Ultraorthodox Jewish communities had consistently lower vaccine uptake across doses than the general Jewish population, with larger gaps in doses 3–4 than in doses 1–2. Differences between the Arab and general Jewish populations were modest for the primary doses, but became pronounced for booster doses. Higher residential SES was consistently associated with greater vaccine uptake, particularly for dose 4, while the peripherality index was significant only for dose 4 uptake. Booster-related disparities remained across age groups, though gaps narrowed among adults ≥ 60 years.

The climate during the initial vaccine rollout (doses 1–2) differed from that during the booster period, in terms of risk perceptions, strict lockdowns, extensive public awareness campaigns, and the implementation of the Green Pass policy, which likely contributed to high compliance with vaccination recommendations across all groups [20]. Additionally, there were dedicated governmental task forces targeting Arab and ultraorthodox Jewish communities, including outreach programs and community-specific vaccine centers [9, 38]. The persistent lower COVID-19 vaccine uptake across all doses in the ultraorthodox Jewish population is not surprising, as this sub-population tends to display vaccine hesitancy, a phenomenon documented in Israel and other sister communities in the United States and Europe [34, 50–52]. The ultraorthodox Jewish population is characterized by a religious lifestyle, involvement of religious leaders (rabbis), specific religious views, lower trust in authorities and social environment, alongside personal attitudes and perceptions that influence the individual’s decision-making regarding vaccination [34, 50–54].

The widening gap in COVID-19 vaccine uptake for booster doses across population groups. and SES, likely reflects declining risk perceptions with booster vaccination deemed unnecessary due to natural infection, reduced media campaigns, and targeted outreach, or a combination of these factors. We found that, among ultraorthodox adults, COVID-19 vaccination decisions were influenced by perceived disease severity, vaccine effectiveness and safety, trust in religious authorities and rabbis, and the use of community-specific communication channels. The perceived disease severity fluctuated with time, and accordingly, the vaccine acceptance [53].

As the pandemic progressed, resources and outreach efforts in Arab and ultraorthodox communities declined, likely contributing to widening gaps, particularly for the fourth dose. Unlike the rapid, mass-center-based rollout of the primary campaign, the booster phase was integrated into routine HMO services with less media attention and fewer mass sites. Messaging also shifted from urgent, universal appeals to more targeted, risk-based recommendations. Overall, this transition potentially increased access barriers and hesitancy.

The positive association between residential SES and COVID-19 vaccine uptake aligns with studies from Europe and the United States [48, 55–58]. Despite widespread access to vaccination centers in Israel, higher uptake in higher-SES communities may reflect greater health.

A global analysis of COVID-19 vaccine booster dose acceptance showed variation across regions, with Europe exhibiting the highest rates and lower rates in American regions [59]. Hesitancy toward COVID-19 vaccine booster doses was estimated at 12% in a survey conducted across 22 countries [10], while in the United States, booster dose uptake was only 48% among fully vaccinated individuals [13]. In agreement with our study, evidence from Norway also showed larger gaps in booster vaccination uptake between less educated, lower-income groups and more affluent groups, compared with the primary series [12]. A prior analysis from Israel noted increased gaps between the Arab and Jewish populations in the uptake of booster doses; however, it did not assess differences between the ultraorthodox and general population, or geographic inequities [18]. Collectively, our and others’ findings demonstrate gaps in the uptake of COVID-19 booster doses across population groups, SES, and regions, which increased in the booster vaccination era.

Israel’s COVID-19 experience offers key lessons for future emergencies. Strong political commitment, professional leadership, substantial public health investment, particularly in vaccines, and inter-ministerial collaboration were central to the response. Strict measures, including lockdowns, gathering bans, and travel restrictions, though restrictive, were feasible and effective in the early phase before vaccines became available. Expanding testing capacity and establishing robust national surveillance, including variant monitoring, enabled data-driven decision-making and led to the creation of a dedicated health intelligence unit for future preparedness.

Our policy analysis of Israel’s COVID-19 vaccination campaign showed that a rapid, centrally coordinated rollout can achieve high coverage and reduce morbidity, in agreement with prior reports [3, 38, 40]. Major lessons learned from the vaccination campaign include a massive, early investment in vaccine procurement, securing enough doses to vaccinate the majority of the population, strong governance, prioritization, and collaboration across the health system, which enabled efficient logistics (e.g., establishing mass vaccination sites with extended opening hours), real-time monitoring, and mass media coverage and campaigns. Israel’s early adoption of booster doses illustrates an adaptive, evidence-driven policy model with global relevance for future pandemic preparedness.

A major challenge was promoting adherence to public health measures and vaccination among Arab and ultraorthodox communities, including non-pharmaceutical measures and vaccination [9, 38, 40]. Although targeted task forces, culturally tailored outreach, and coordination with community leaders reduced some gaps [40], disparities widened during the booster phase, underscoring persistent equity challenges. Addressing these requires sustained efforts to improve access (e.g., augment staffing, expand vaccination sites), strengthen community partnerships, enhance health literacy, and provide targeted incentives, particularly as attention and resources decline in later phases. As emergencies shift from acute to long-term phases, sustained attention and resources are needed to support existing infrastructure and prevent widening gaps.

Additional areas for improvement remain essential for future preparedness, including strengthening public health infrastructure during outbreaks and pandemics; expanding workforce capacity; implementing electronic/digital systems to rapidly process complex epidemiological data, such as epidemiological investigation and contact tracing; and addressing vaccine side effects, as there were some drawbacks in these areas.

Routine vaccination in Israel is voluntary, and, in general, medical interventions require patients’ informed consent under the Patient’s Rights Law in Israel [60], although in emergency situations, such as pandemics, mandatory vaccination might be considered to increase vaccine uptake and protect the public [61]. However, this sparks debate over public health benefits and individual autonomy, and raises concerns about informed consent and public trust. During the COVID-19 pandemic mandatory vaccination Law was considered but eventually not passed [62]. An incentive model such as Israel’s “Green Pass” [41, 63] may increase vaccine uptake, but it also raises ethical questions and concerns regarding the rights of unvaccinated individuals [41]. Despite the introduction of the Green Pass policy, implementation and enforcement gaps may have occurred [41]. Israel’s experience shows that the Green Pass model was an efficient alternative to mandatory vaccination, balancing between public health and the individual’s autonomy, and can be better implemented in future pandemics.

Our study has limitations. The population group was classified by the predominant ethnicity/group in each town, which does not capture the within-city heterogeneity. We analyzed vaccine dose counts, without accounting for naturally acquired or hybrid immunity. We limited the analysis to towns with ≥ 10,000 residents to allow stratified analyses, which might limit generalizability to smaller communities.

Our study has several strengths. It is based on a nationwide analysis of a large dataset comprising 135 towns with diverse population groups, SES strata, and geographic regions, enabling a comprehensive assessment of vaccination trends in Israel across both the primary series and booster vaccination phases. We examined key community-level social determinants, including residential SES, peripherality, and population groups across age groups, providing detailed insights into vaccine hesitancy patterns. The study’s longitudinal perspective captures changes over time, and the accompanying policy analysis offers practical recommendations to address vaccine inequities currently and in future emergencies.

## Conclusions

This nationwide analysis demonstrates persistent and widening disparities in COVID-19 vaccine uptake in Israel across population groups and socioeconomic strata, particularly during the booster phase. Ultraorthodox Jewish and Arab communities and those from lower-SES populations consistently exhibited lower uptake, with gaps widening for booster doses, as well as living in peripheral regions, which was linked with lower uptake of the fourth dose. Collectively, these findings suggest the emergence of barriers to vaccine access and hesitancy. While Israel’s rapid, centrally coordinated primary vaccination campaign achieved high overall coverage, sustaining equitable uptake proved more challenging as the pandemic evolved and vaccination shifted into a routine phase. These findings underscore that successful emergency deployment alone is insufficient to ensure long-term equity. Maintaining targeted outreach, culturally tailored communication, accessible vaccination infrastructure, and sustained public engagement is essential, especially during later stages when public attention wanes and risk perception may change. Israel’s experience highlights the importance of strong governance, early investment, real-time data systems, and adaptive evidence-based policy-making, while also illustrating the ethical and practical complexities of balancing public health goals with individual autonomy through incentive-based models such as the Green Pass. Future pandemic preparedness strategies must proactively integrate sustained equity-focused approaches to prevent widening disparities, especially in the longer term, and ensure inclusive protection across all population groups.

## Supplementary Information

Additional file 1.

Additional file 2.

Additional file 3.

Additional file 4.

## Data Availability

The data used in this study are publicly available at Israel’s Ministry Website at: [https://data.gov.il/dataset/covid-19]. (https:/data.gov.il/dataset/covid-19). The source of the sociodemographic data is the Israel Central Bureau of Statistics, data available from: https://www.cbs.gov.il/he/mediarelease/pages/2021/%D7%99%D7%A9%D7%A8%D7%90%D7%9C-%D7%91%D7%9E%D7%A1%D7%A4%D7%A8%D7%99%D7%9D-%D7%A2%D7%A8%D7%91-%D7%A8%D7%90%D7%A9-%D7%94%D7%A9%D7%A0%D7%94-%D7%AA%D7%A9%D7%A4%D7%91-%D7%A0%D7%AA%D7%95%D7%A0%D7%99%D7%9D-%D7%A0%D7%91%D7%97%D7%A8%D7%99%D7%9D-%D7%9E%D7%94%D7%9C%D7%95%D7%97%D7%95%D7%AA-%D7%94%D7%A9%D7%A0%D7%AA%D7%99%D7%99%D7%9D-2021.aspx

## Abbreviations

CI: Confidence interval
COVID-19: Coronavirus disease 2019
HMO: Health maintenance organizations
ICBS: Israel Central Bureau of Statistics
IRR: Incident rate ratio
MOH: Ministry of Health
SARS-CoV-2: Severe acute respiratory syndrome coronavirus 2
SES: Socioeconomic status

## Footnotes

1 The “Green Pass” was a government-issued certification system introduced during the COVID-19 pandemic that granted vaccinated or recovered (immune) individuals access to specified public venues, including cultural, social, and sporting events. This certificate served as an incentive to promote vaccination and facilitate the safe reopening of economic and social activities. Unvaccinated individuals were requested to present valid negative COVID-19 test results.

2 Support tests are guidelines that define the criteria for allocating state budget funds to public institutions. Each type of institution or field of activity is assigned a budget regulation specifying the total amount of support. By law, this support must be distributed equitably according to tests determined by the responsible minister, in consultation with and approved by the Attorney General.

## References

[1] WHO COVID-19 dashboard. https://data.who.int/dashboards/covid19/deaths?n=o

[2] Muhsen K, Cohen D. COVID-19 vaccination in Israel. Clin Microbiol Infect. 2021;27(11):1570–4. 34384875 10.1016/j.cmi.2021.07.041 PMC8351306

[3] Rosen B, Waitzberg R, Israeli A. Israel’s rapid rollout of vaccinations for COVID-19. Isr J Health Policy Res. 2021;10(1):6. 33499905 10.1186/s13584-021-00440-6 PMC7835664

[4] Corona dashboard - Ministry of Health. https://datadashboard.health.gov.il/portal/dashboard/corona

[5] Bar-On YM, Goldberg Y, Mandel M, Bodenheimer O, Freedman L, Alroy-Preis S, et al. Protection against Covid-19 by BNT162b2 booster across age groups. N Engl J Med. 2021;385(26):2421–30. 34879188 10.1056/NEJMoa2115926 PMC8728796

[6] Muhsen K, Maimon N, Mizrahi AY, Varticovschi B, Bodenheimer O, Cohen D, et al. Association of BNT162b2 vaccine third dose receipt with incidence of SARS-CoV-2 infection, COVID-19-related hospitalization, and death among residents of long-term care facilities, August to October 2021. JAMA Netw Open. 2022;5(7):e2219940. 35796153 10.1001/jamanetworkopen.2022.19940 PMC9250055

[7] Bar-On YM, Goldberg Y, Mandel M, Bodenheimer O, Amir O, Freedman L, et al. Protection by a fourth dose of BNT162b2 against Omicron in Israel. N Engl J Med. 2022;386(18):1712–20. 35381126 10.1056/NEJMoa2201570 PMC9006780

[8] Muhsen K, Maimon N, Mizrahi AY, Boltyansky B, Bodenheimer O, Diamant ZH, et al. Association of receipt of the fourth BNT162b2 dose with Omicron infection and COVID-19 hospitalizations among residents of long-term care facilities. JAMA Intern Med. 2022;182(8):859–67. 35737368 10.1001/jamainternmed.2022.2658 PMC9227688

[9] Muhsen K, Na’aminh W, Lapidot Y, Goren S, Amir Y, Perlman S, et al. A nationwide analysis of population group differences in the COVID-19 epidemic in Israel, February 2020-February 2021. Lancet Reg Health Eur. 2021;7:100130. 34109321 10.1016/j.lanepe.2021.100130 PMC8177966

[10] Lazarus JV, Wyka K, White TM, Picchio CA, Gostin LO, Larson HJ, et al. A survey of COVID-19 vaccine acceptance across 23 countries in 2022. Nat Med. 2023;29(2):366–75. 36624316 10.1038/s41591-022-02185-4

[11] Wang H, Varol T, Gültzow T, Zimmermann HML, Ruiter RAC, Jonas KJ. Spatio-temporal distributions of COVID-19 vaccine doses uptake in the Netherlands: a Bayesian ecological modelling analysis. Epidemiol Infect. 2024;152:e119. 39370683 10.1017/S0950268824001249 PMC11474892

[12] Hansen BT, Labberton AS, Kour P, Kraft KB. Coverage of primary and booster vaccination against COVID-19 by socioeconomic level: a nationwide cross-sectional registry study. Hum Vaccin Immunother. 2023;19(1):2188857. 36941785 10.1080/21645515.2023.2188857 PMC10072069

[13] Agaku IT, Adeoye C, Long TG. Geographic, occupational, and sociodemographic variations in uptake of COVID-19 booster doses among fully vaccinated US adults, December 1, 2021, to January 10, 2022. JAMA Netw Open. 2022;5(8):e2227680. 35984657 10.1001/jamanetworkopen.2022.27680 PMC9391956

[14] Wood AJ, MacKintosh AM, Stead M, Kao RR. Long-term spatial patterns in COVID-19 booster vaccine uptake. Commun Med. 2025;5(1):257. 40596654 10.1038/s43856-025-00949-w PMC12215375

[15] Ber I, Lerman Y, Muhsen K. The need for reducing disparities in SARS-COV-2 immunization: the Ultraorthodox and Arab populations in Israel. Harefuah. 2021;160(5):285–90. 34028219

[16] Benderly M, Huppert A, Novikov I, Ziv A, Kalter-Leibovici O. Fighting a pandemic: sociodemographic disparities and coronavirus disease-2019 vaccination gaps-a population study. Int J Epidemiol. 2022;51(3):709–17. 35104860 10.1093/ije/dyac007 PMC8903338

[17] Gorelik Y, Anis E, Edelstein M. Inequalities in initiation of COVID19 vaccination by age and population group in Israel- December 2020-July 2021. Lancet Reg Health Eur. 2022;12:100234. 34746908 10.1016/j.lanepe.2021.100234 PMC8556198

[18] Luxenburg O, Singer C, Kaim A, Saban M, Wilf-Miron R. Socioeconomic and ethnic disparities along five waves of the COVID-19 pandemic: lessons we have not yet learnt. J Nurs Scholarsh. 2023;55(1):45–55. 36218245 10.1111/jnu.12816 PMC9874613

[19] Rosen B, Hartal M, Waitzberg R. The Israeli health system’s rapid responses during the COVID-19 pandemic. Isr J Health Policy Res. 2024;13(1):11. 38438926 10.1186/s13584-024-00596-x PMC10910866

[20] Ash N, Triki N, Waitzberg R. The COVID-19 pandemic posed many dilemmas for policymakers, which sometimes resulted in unprecedented decision-making. Isr J Health Policy Res. 2023;12(1):13. 37072814 10.1186/s13584-023-00564-x PMC10112313

[21] Israel Central Bureau of Statistics. Statistical Abstract of Israel 2022. Israel Central Bureau of Statistics; State of Israel. 2022;73

[22] Malach G, Cahaner L. Statistical report on ultraorthodox society in Israel. The Israel Democracy Institute. 2022.

[23] Israel Central Bureau of Statistics. Characterization and classification of geographical units by the socio-economic level of the population, 2021. Israel Central Bureau of Statistics, State of Israel. 2022.

[24] Shalizi C. Distances between clustering, hierarchical clustering 2009. [cited 2025 Apr 27]. Available from: https://www.stat.cmu.edu/~cshalizi/350/lectures/08/lecture-08.pdf

[25] Birenbaum-Carmeli D, Chassida J. Covid-19 in Israel: socio-demographic characteristics of first wave morbidity in Jewish and Arab communities. Int J Equity Health. 2020;19(1):153. 32907584 10.1186/s12939-020-01269-2 PMC7480661

[26] COVID-19 dataset – settlements table. https://data.gov.il/dataset/covid-19/resource/8a21d39d-91e3-40db-aca1-f73f7ab1df69

[27] COVID-19 dataset – vaccinated by settlement. https://data.gov.il/dataset/covid-19/resource/12c9045c-1bf4-478a-a9e1-1e876cc2e182

[28] Malach G, Cahaner L. The yearbook of the ultraorthodox sector. Chapter 1: population**.** The Israel Democracy Institute. 2022.

[29] The Haredi Institute for Policy Research. Demographic data of the Haredi community in Israel. [cited 2025 Jan 19]. Available from: https://data.machon.org.il/dashbords/demography/

[30] Israel Central Bureau of Statistics. Peripherality index of localities and local authorities, 2015. Israel Central Bureau of Statistics; State of Israel. 2019.

[31] Walt G, Gilson L. Reforming the health sector in developing countries: the central role of policy analysis. Health Policy Plan. 1994;9(4):353–70. 10139469 10.1093/heapol/9.4.353

[32] Clarfield AM, Manor O, Nun GB, Shvarts S, Azzam ZS, Afek A, et al. Health and health care in Israel: an introduction. Lancet. 2017;389(10088):2503–13. 28495109 10.1016/S0140-6736(17)30636-0

[33] Balicer RD, Afek A. Digital health nation: Israel’s global big data innovation hub. The Lancet. 2017;389(10088):2451–3. 10.1016/S0140-6736(17)30876-0 28495105

[34] Jacobson A, Spitzer S, Gorelik Y, Edelstein M. Barriers and enablers to vaccination in the ultra-orthodox Jewish population: a systematic review. Front Public Health. 2023;11:1244368. 37900036 10.3389/fpubh.2023.1244368 PMC10602685

[35] Israel Ministry of Health. Vaccination coverage of routine childhood vaccination 2017-2022 in Israel**.** 2023.

[36] Shitrit AVB, Huppert A. Health disparities: influenza vaccination. In: Internet.

[37] State Comptroller of Israel. Vaccination of the population against the Coronavirus. State Comptroller of Israel 2024.

[38] Muhsen K, Cohen D, Glatman-Freedman A, Husseini S, Perlman S, McNeil C. Review of Israel’s action and response during the COVID-19 pandemic and tabletop exercise for the evaluation of readiness and resilience-lessons learned 2020-2021. Front Public Health. 2023;11:1308267. 38328537 10.3389/fpubh.2023.1308267 PMC10847317

[39] Muhsen K, Maimon N, Mizrahi A, Bodenheimer O, Cohen D, Maimon M, et al. Effectiveness of BNT162b2 mRNA Coronavirus Disease 2019 (COVID-19) vaccine against acquisition of severe Acute Respiratory Syndrome Coronavirus 2 (SARS-CoV-2) among healthcare workers in long-term care facilities: a prospective cohort study. Clin Infect Dis. 2022;75(1):e755–63. 34698808 10.1093/cid/ciab918 PMC8675294

[40] Rosen B, Waitzberg R, Israeli A, Hartal M, Davidovitch N. Addressing vaccine hesitancy and access barriers to achieve persistent progress in Israel’s COVID-19 vaccination program. Isr J Health Policy Res. 2021;10(1):43. 34340714 10.1186/s13584-021-00481-x PMC8326649

[41] Waitzberg R, Triki N, Alroy-Preis S, Lotan T, Shiran L, Ash N. The Israeli experience with the “Green Pass” policy highlights issues to נe considered by policymakers in other countries. Int J Environ Res Public Health. 2021. 10.3390/ijerph182111212. 34769731 10.3390/ijerph182111212 PMC8582817

[42] Bar-On YM, Goldberg Y, Mandel M, Bodenheimer O, Freedman L, Kalkstein N, et al. Protection of BNT162b2 vaccine booster against Covid-19 in Israel. N Engl J Med. 2021;385(15):1393–400. 34525275 10.1056/NEJMoa2114255 PMC8461568

[43] Haas EJ, McLaughlin JM, Khan F, Angulo FJ, Anis E, Lipsitch M, et al. Infections, hospitalisations, and deaths averted via a nationwide vaccination campaign using the Pfizer-BioNTech BNT162b2 mRNA COVID-19 vaccine in Israel: a retrospective surveillance study. Lancet Infect Dis. 2022;22(3):357–66. 34562375 10.1016/S1473-3099(21)00566-1 PMC8457761

[44] Mevorach D, Anis E, Cedar N, Bromberg M, Haas EJ, Nadir E, et al. Myocarditis after BNT162b2 mRNA vaccine against Covid-19 in Israel. N Engl J Med. 2021;385(23):2140–9. 34614328 10.1056/NEJMoa2109730 PMC8531987

[45] Efrati I. The national vaccination campaign started. In: Haaretz. 2020.

[46] Israel Ministry of Health: HMOs roll out an Omicron-specific vaccine**.** https://www.gov.il/en/pages/20092022-04

[47] Hilae S. Hundreds are getting vaccinated at Dizengoff Square in Ynet: Ynet; 2021.

[48] Gaughan CH, Razieh C, Khunti K, Banerjee A, Chudasama YV, Davies MJ, et al. COVID-19 vaccination uptake amongst ethnic minority communities in England: a linked study exploring the drivers of differential vaccination rates. J Public Health (Oxf). 2023;45(1):e65–74. 34994801 10.1093/pubmed/fdab400 PMC8755382

[49] Gesser-Edelsburg A, Hijazi R, Cohen R. It takes two to tango: How the COVID-19 vaccination campaign in Israel was framed by the Health Ministry vs. the television news. Front Public Health. 2022;10:887579. 35493372 10.3389/fpubh.2022.887579 PMC9039239

[50] Carmody ER, Zander D, Klein EJ, Mulligan MJ, Caplan AL. Knowledge and attitudes toward Covid-19 and vaccines among a New York Haredi-Orthodox Jewish community. J Community Health. 2021;46(6):1161–9. 33999317 10.1007/s10900-021-00995-0 PMC8127857

[51] Berger Z. Health system mistrust, Ultra-Orthodox Jews in the US, and vaccine hesitancy. J Biosoc Sci. 2025;57(2):195–200. 40062766 10.1017/S0021932025000124

[52] Shapiro E. Orthodox Jews and factors affecting COVID-19 vaccination: Lessons learned for public health. Eur J Public Health. 2023. 10.1093/eurpub/ckad160.913.

[53] Ber I, Na’amnih W, Perlman S, Kasstan B, Lerman Y, Muhsen K. Developing and validating a culturally tailored questionnaire to assess COVID-19 vaccine hesitancy in Israel’s Ultraorthodox Jewish population. Hum Vaccin Immunother. 2024;20(1):2429233. 39635713 10.1080/21645515.2024.2429233 PMC11622615

[54] Gendler Y, Ofri L, Videl H. Understanding factors contributing to vaccination underutilization among Jewish Ultra-Orthodox communities in Israel: a cross-sectional study. Vaccine. 2025;47:126711. 39793538 10.1016/j.vaccine.2025.126711

[55] Topmiller M, Carrozza M, McCann J, Rankin J. Identifying health centers in areas with low COVID-19 vaccination rates & high rates of vaccine hesitancy. Ann Fam Med. 2022. 10.1370/afm.20.s1.2734. 36706265 10.1370/afm.20.s1.2734 PMC10548910

[56] Lacy A, Khan MM, Deb Nath N, Das P, Igoe M, Lenhart S, et al. Geographic disparities and predictors of COVID-19 vaccination in Missouri: a retrospective ecological study. Front Public Health. 2024;12:1329382. 38528866 10.3389/fpubh.2024.1329382 PMC10961407

[57] Chen H, Cao Y, Feng L, Zhao Q, Torres JRV. Understanding the spatial heterogeneity of COVID-19 vaccination uptake in England. BMC Public Health. 2023;23(1):895. 37189026 10.1186/s12889-023-15801-w PMC10185460

[58] Peña JM, Schwartz MR, Hernandez-Vallant A, Sanchez GR. Social and structural determinants of COVID-19 vaccine uptake among racial and ethnic groups. J Behav Med. 2023;46(1–2):129–39. 36652085 10.1007/s10865-023-00393-y PMC9846662

[59] Roy DN, Ferdiousi N, Mohabbot Hossen M, Islam E, Shah Azam M. Global disparities in COVID-19 vaccine booster dose (VBD) acceptance and hesitancy: an updated narrative review. Vaccine X. 2024;18:100480. 38585380 10.1016/j.jvacx.2024.100480 PMC10997838

[60] Patient’s Rights Law in Israel 1996. https://www.nevo.co.il/law_html/law00/71833.htm

[61] Public Health Ordinance No. 40 of 1940 Section 20 (2). https://www.nevo.co.il/law_html/law01/049_001.htm#Seif21

[62] Saban M, Myers V, Ben Shetrit S, Wilf-Miron R. Issues surrounding incentives and penalties for COVID-19 vaccination: the Israeli experience. Prev Med. 2021;153:106763. 34352308 10.1016/j.ypmed.2021.106763 PMC8327565

[63] Kamin-Friedman S, Peled Raz M. Lessons from Israel’s COVID-19 Green Pass program. Isr J Health Policy Res. 2021;10(1):61. 34715931 10.1186/s13584-021-00496-4 PMC8554179

