# Supplementary material for: Trends in socio-demographic disparities in COVID-19 vaccine uptake by vaccine dose and time after the introduction of COVID-19 vaccination in Israel: epidemiological and policy analysis study
Source: Isr J Health Policy Res. 2026 May 4;15:15. doi: 10.1186/s13584-026-00758-z (PMC13137699; doi:10.1186/s13584-026-00758-z)
Supplement: Supplementary file 1 — Additional file 1. [file 13584_2026_758_MOESM1_ESM.pdf]

Additional file 4

Bivariate analysis

In bivariate analysis, significant differences were found in vaccine uptake across all doses, in the combined analysis of all ages, in each study period, and as well as in each age group/period separately. Specifically, the COVID-19 vaccine uptake was lower in the Arab and ultraorthodox Jewish populations compared to the general Jewish population. A positive association was found between SES rank and vaccine uptake, as well as between peripherality index and vaccine uptake (Supplementary Tables 3-6).

Supplementary Table 3: Bivariate analysis of residential sociodemographic factors and COVID-19 vaccine dose 1 uptake by age and period

	**Uptake of COVID-19 vaccine – dose 1-all ages**		**Uptake of COVID-19 vaccine – dose 1-age 20-29 years**		**Uptake of COVID-19 vaccine – dose 1-age 30-59 years**		**Uptake of COVID-19 vaccine – dose 1–age ≥60 years**	
	**IRR (95% CI)**	**P value**	**IRR (95% CI)**	**P value**	**IRR (95% CI)**	**P value**	**IRR (95% CI)**	**P value**
**Period 1**								
Population group		<0.001		<0.001		<0.001		<0.001
General Jewish population towns	Reference		Reference		Reference		Reference	
Ultraorthodox towns	0.617 (0.615–0.619)	<0.001	0.743 (0.738–0.747)	<0.001	0.787 (0.783–0.790)	<0.001	0.915 (0.910–0.920)	<0.001
Arab towns	0.738 (0.736–0.740)	<0.001	0.843 (0.838–0.848)	<0.001	0.856 (0.853–0.860)	<0.001	0.818 (0.812–0.824)	<0.001
SES rank	1.087 (1.087-1.087)	<0.001	1.058 (1.057–1.059)	<0.001	1.042 (1.041–1.042)	<0.001	1.025 (1.024–1.026)	<0.001
Peripherality index	1.019 (1.018-1.019)	<0.001	1.009 (1.008–1.010)	<0.001	1.005 (1.005–1.006)	<0.001	1.012 (1.012–1.013)	<0.001
**Period 2**								
Population group		<0.001		<0.001		<0.001		<0.001
General Jewish population towns	Reference		Reference		Reference		Reference	
Ultraorthodox towns	0.689 (0.687–0.690)	<0.001	0.822 (0.818–0.826)	<0.001	0.846 (0.843–0.849)	<0.001	0.934 (0.929–0.939)	<0.001
Arab towns	0.798 (0.795–0.800)	<0.001	0.891 (0.886–0.896)	<0.001	0.896 (0.892–0.899)	<0.001	0.837 (0.831–0.843)	<0.001
SES rank	1.070 (1.070-1.071)	<0.001	1.039 (1.038–1.040)	<0.001	1.029 (1.029–1.030)	<0.001	1.020 (1.019–1.021)	<0.001
Peripherality index	1.016 (1.015-1.016)	<0.001	1.007 (1.006–1.008)	<0.001	1.004 (1.003–1.004)	<0.001	1.011 (1.011–1.012)	<0.001
**Period 3**								
Population group		<0.001		<0.001		<0.001		<0.001
General Jewish population towns	Reference		Reference		Reference		Reference	
Ultraorthodox towns	0.696 (0.694–0.698)	<0.001	0.827 (0.823–0.831)	<0.001	1.453 (1.446–1.461)	<0.001	0.935 (0.930–0.941)	<0.001
Arab towns	0.796 (0.794–0.799)	<0.001	0.895 (0.890–0.900)	<0.001	1.447 (1.439–1.455)	<0.001	0.838 (0.832–0.844)	<0.001
SES rank	1.069 (1.069-1.070)	<0.001	1.038 (1.037–1.039)	<0.001	1.029 (1.028–1.029)	<0.001	1.020 (1.019–1.021)	<0.001
Peripherality index	1.016 (1.016-1.017)	<0.001	1.007 (1.006–1.008)	<0.001	1.004 (1.003–1.004)	<0.001	1.011 (1.011–1.012)	<0.001

CI: confidence interval; COVID-19: coronavirus disease 2019; IRR: Incident rate ratio; SES: socioeconomic status.

Supplementary Table 4: Univariate analysis of residential sociodemographic factors and COVID-19 vaccine dose 2 uptake by age and period

	**Uptake of COVID-19 vaccine – dose 2- all ages**		**Uptake of COVID-19 vaccine – dose 2- age 20-29 years**		**Uptake of COVID-19 vaccine – dose 2- age 30-59 years**		**Uptake of COVID-19 vaccine – dose 2– age ≥60 years**	
	**IRR (95% CI)**	**P value**	**IRR (95% CI)**	**P value**	**IRR (95% CI)**	**P value**	**IRR (95% CI)**	**P value**
**Period 1**								
Population group		<0.001		<0.001		<0.001		<0.001
General Jewish population towns	Reference		Reference		Reference		Reference	
Ultraorthodox towns	0.573 (0.572–0.575)	<0.001	0.685 (0.681–0.689)	<0.001	0.729 (0.726–0.732)	<0.001	0.871 (0.866–0.876)	<0.001
Arab towns	0.704 (0.702–0.706)	<0.001	0.796 (0.791–0.801)	<0.001	0.817 (0.813–0.820)	<0.001	0.774 (0.768–0.781)	<0.001
SES rank	1.098 (1.098-1.099)	<0.001	1.073 (1.072–1.074)	<0.001	1.054 (1.054–1.055)	<0.001	1.034 (1.033–1.035)	<0.001
Peripherality index	1.019 (1.019-1.020)	<0.001	1.010 (1.009–1.011)	<0.001	1.007 (1.006–1.008)	<0.001	1.013 (1.012–1.014)	<0.001
**Period 2**								
Population group		<0.001		<0.001		<0.001		<0.001
General Jewish population towns	Reference		Reference		Reference		Reference	
Ultraorthodox towns	0.610 (0.609–0.612)	<0.001	0.721 (0.717–0.726)	<0.001	0.760 (0.757–0.763)	<0.001	0.895 (0.890–0.900)	<0.001
Arab towns	0.730 (0.728–0.732)	<0.001	0.835 (0.830–0.840)	<0.001	0.831 (0.828–0.835)	<0.001	0.785 (0.779–0.791)	<0.001
SES rank	1.091 (1.090-1.091)	<0.001	1.063 (1.062–1.063)	<0.001	1.048 (1.047–1.049)	<0.001	1.029 (1.028–1.030)	<0.001
Peripherality index	1.019 (1.018-1.019)	<0.001	1.008 (1.007–1.009)	<0.001	1.007 (1.006–1.008)	<0.001	1.013 (1.012–1.014)	<0.001
**Period 3**								
Population group		<0.001		<0.001		<0.001		<0.001
General Jewish population towns	Reference		Reference		Reference		Reference	
Ultraorthodox towns	0.610 (0.608–0.612)	<0.001	0.729 (0.725–0.733)	<0.001	0.767 (0.764–0.770)	<0.001	0.901 (0.896–0.906)	<0.001
Arab towns	0.722 (0.720–0.724)	<0.001	0.839 (0.834–0.844)	<0.001	0.835 (0.831–0.838)	<0.001	0.791 (0.785–0.797)	<0.001
SES rank	1.093 (1.092-1.093)	<0.001	1.061 (1.060–1.062)	<0.001	1.047 (1.046–1.047)	<0.001	1.028 (1.027–1.029)	<0.001
Peripherality index	1.020 (1.020-1.020)	<0.001	1.008 (1.007–1.009)	<0.001	1.007 (1.007–1.008)	<0.001	1.012 (1.012–1.013)	<0.001

CI: confidence interval; COVID-19: coronavirus disease 2019; IRR: Incident rate ratio; SES: socioeconomic status.

Supplementary Table 5: Univariate analysis of residential sociodemographic factors and COVID-19 vaccine dose 3 uptake by age and period

	**Uptake of COVID-19 vaccine – dose 3- all ages**		**Uptake of COVID-19 vaccine – dose 3- age 20-29 years**		**Uptake of COVID-19 vaccine – dose 3- age 30-59 years**		**Uptake of COVID-19 vaccine – dose 3– age ≥60 years**	
	**IRR (95% CI)**	**P value**	**IRR (95% CI)**	**P value**	**IRR (95% CI)**	**P value**	**IRR (95% CI)**	**P value**
**Period 2**								
Population group		<0.001		<0.001		<0.001		<0.001
General Jewish population towns	Reference		Reference		Reference		Reference	
Ultraorthodox towns	0.484 (0.483–0.486)	<0.001	0.551 (0.547–0.555)	<0.001	0.638 (0.635–0.641)	<0.001	0.832 (0.827–0.837)	<0.001
Arab towns	0.525 (0.523–0.527)	<0.001	0.620 (0.615–0.625)	<0.001	0.632 (0.628–0.635)	<0.001	0.657 (0.652–0.663)	<0.001
SES rank	1.145 (1.145-1.146)	<0.001	1.130 (1.129–1.131)	<0.001	1.096 (1.096–1.097)	<0.001	1.051 (1.050–1.052)	<0.001
Peripherality index	1.036 (1.036-1.037)	<0.001	1.023 (1.022–1.024)	<0.001	1.025 (1.024–1.025)	<0.001	1.018 (1.017–1.019)	<0.001
**Period 3**								
Population group		<0.001		<0.001		<0.001		<0.001
General Jewish population towns	Reference		Reference		Reference		Reference	
Ultraorthodox towns	0.491 (0.490–0.493)	<0.001	0.572 (0.568–0.576)	<0.001	0.646 (0.643–0.649)	<0.001	0.839 (0.834–0.844)	<0.001
Arab towns	0.538 (0.536–0.540)	<0.001	0.656 (0.651–0.661)	<0.001	0.648 (0.644–0.651)	<0.001	0.661 (0.656–0.667)	<0.001
SES rank	1.142 (1.142-1.143)	<0.001	1.121 (1.119–1.122)	<0.001	1.093 (1.092–1.093)	<0.001	1.048 (1.048–1.049)	<0.001
Peripherality index	1.035 (1.035-1.036)	<0.001	1.020 (1.019–1.021)	<0.001	1.023 (1.023–1.024)	<0.001	1.018 (1.017–1.019)	<0.001

CI: confidence interval; COVID-19: coronavirus disease 2019; IRR: Incident rate ratio; SES: socioeconomic statu

Supplementary Table 6: Univariate analysis of residential sociodemographic factors and COVID-19 vaccine dose 4 uptake by age and period

	**Uptake of COVID-19 vaccine – dose 4- all ages**		**Uptake of COVID-19 vaccine – dose 4- age 20-29 years**		**Uptake of COVID-19 vaccine – dose 4- age 30-59 years**		**Uptake of COVID-19 vaccine – dose 4– age ≥60 years**	
	**IRR (95% CI)**	**P value**	**IRR (95% CI)**	**P value**	**IRR (95% CI)**	**P value**	**IRR (95% CI)**	**P value**
**Period 3**								
Population group		<0.001		<0.001		<0.001		<0.001
General Jewish population towns	Reference		Reference		Reference		Reference	
Ultraorthodox towns	0.243 (0.126–0.471)	<0.001	0.358 (0.183–0.702)	<0.001	0.255 (0.131–0.493)	<0.001	0.240 (0.124–0.464)	<0.001
Arab towns	0.155 (0.108–0.222)	<0.001	0.519 (0.358–0.751)	<0.001	0.263 (0.183–0.377)	<0.001	0.131 (0.092–0.188)	<0.001
SES rank	1.454 (1.338–1.580)	<0.001	1.193 (1.109–1.282)	<0.001	1.356 (1.256–1.465)	<0.001	1.489 (1.368–1.622)	<0.001
Peripherality index	1.178 (1.092–1.272)	<0.001	1.097 (1.011–1.190)	<0.001	1.159 (1.072–1.252)	<0.001	1.184 (1.097–1.277)	<0.001

CI: confidence interval; COVID-19: coronavirus disease 2019; IRR: Incident rate ratio; SES: socioeconomic status.
