# Supplementary material for: Trends in socio-demographic disparities in COVID-19 vaccine uptake by vaccine dose and time after the introduction of COVID-19 vaccination in Israel: epidemiological and policy analysis study
Source: Isr J Health Policy Res. 2026 May 4;15:15. doi: 10.1186/s13584-026-00758-z (PMC13137699; doi:10.1186/s13584-026-00758-z)
Supplement: Supplementary file 2 — Additional file 2. [file 13584_2026_758_MOESM2_ESM.pdf]

Supplementary Figure 1: Correlation matrix of independent variables

	**Population group**	**Average monthly salary – employees**	**Average monthly salary – self-employed**	**Residential socioeconomic rank**	**Peripherality index**
**Average monthly salary – employees**	**-0.809**				
**Average monthly salary – self-employed**	**-0.543**	**0.729**			
**Residential socioeconomic rank**	**-0.764**	**0.959**	**0.722**		
**Peripherality index**	**-0.454**	**0.543**	**0.424**	**0.557**	
**Population density**	**-0.325**	**0.242**	0.193	**0.249**	**0.706**

The data presented is Spearman's correlation coefficient

Bold number bold numbers represent significant results ($p<0.05$). orange/red cells represent negative correlations, and green/yellow cells represent positive correlations.
