# Supplementary material for: Trends in socio-demographic disparities in COVID-19 vaccine uptake by vaccine dose and time after the introduction of COVID-19 vaccination in Israel: epidemiological and policy analysis study
Source: Isr J Health Policy Res. 2026 May 4;15:15. doi: 10.1186/s13584-026-00758-z (PMC13137699; doi:10.1186/s13584-026-00758-z)
Supplement: Supplementary file 3 — Additional file 3. [file 13584_2026_758_MOESM3_ESM.pdf]

Supplementary Table 1: Population-based analysis of SARS-CoV-2 infection rates and testing uptake by ethnicity and period

Variable	Overall	General Jewish population	Ultraorthodox Jewish population	Arab population
**Number of towns**	135	76	10	49
**Rate of SARS-CoV-2 infection, per 10,000 residents, median (IQR)**				
Period 1, June 30, 2021	795 (512)	617 (293)	1,881 (916)	937 (264)
Min-max	260 -3,018	301 - 1,583	1,256 - 3,018	260 - 1,554
Period 2, December 31, 2021	1,407 (609)	1,207 (466)	2,365 (1,125)	1,568 (345)
Min-max	663 - 3,598	663 - 2,495	1,712 - 3,598	762 - 1,990
Period 3, December 31, 2022	5,081 (1,392)	5,499 (571)	4,375 (1,250)	4,097 (915)
Min-max	1,552 - 6,609	3,833 - 6,609	3,064 - 5,320	1,552 - 5,289
**Rate of uptake of SARS-CoV-2 diagnostic test, per 10,000 residents, median (IQR)**				
Period 1, June 30, 2021	17,045 (5,643)	18,075 (3,097)	16,237 (6,211)	12,319 (4,121)
Min-max	4,408 - 33,199	11,315 - 33,199	11,564 - 20,135	4,408 - 20,296
Period 2, December 31, 2021	34,768 (11,774)	37,914 (7,465)	25,936 (5,664)	27,267 (8,609)
Min-max	11,973 - 63,508	25,609 - 63,508	17,185 - 34,175	11,973 - 41,095
Period 3, December 31, 2022	57,622 (19,920)	62,949 (12,441)	35,289 (14,658)	44,740 (15,489)
Min-max	18,948 - 93,410	38,979 - 93,410	20,960 - 47,468	18,948 - 69,746

SARS-CoV-2-severe acute respiratory syndrome coronavirus 2, IQR-interquartile range, Min-minimum, Max-maximum.
