# Supplementary material for: Trends in socio-demographic disparities in COVID-19 vaccine uptake by vaccine dose and time after the introduction of COVID-19 vaccination in Israel: epidemiological and policy analysis study
Source: Isr J Health Policy Res. 2026 May 4;15:15. doi: 10.1186/s13584-026-00758-z (PMC13137699; doi:10.1186/s13584-026-00758-z)
Supplement: Supplementary file 4 — Additional file 4. [file 13584_2026_758_MOESM4_ESM.pdf]

Supplementary Table 2: Adjusted incidence rate ratios (IRR) of confirmed SARS-CoV-2 infections by population group and socioeconomic factors by study periods

Variable	Overall rate of confirmed SARS-CoV-2 infection		Overall rate of confirmed SARS-CoV-2 infection		Overall rate of confirmed SARS-CoV-2 infection	
	Period-1		Period-2		Period-3	
	IRR (95% CI)	P value	IRR (95% CI)	P value	IRR (95% CI)	P value
**Population group**		0.004		0.150		0.162
General Jewish population towns	Reference		Reference		Reference	
Ultraorthodox towns	2.79 (1.44-5.41)	0.002	1.88 (0.97-3.64)	0.059	0.79 (0.41-1.54)	0.503
Arab towns	1.40 (0.97-2.00)	0.065	1.17 (0.82-1.68)	0.379	0.70 (0.49-1.01)	0.059
**SES rank**	0.88 (0.81-0.95)	0.001	0.92 (0.85-0.99)	0.043	1.07 (1.00-1.16)	0.051
**Peripherality index**	0.98 (0.90, 1.06)	0.630	0.98 (0.91-1.07)	0.748	1.03 (0.95-1.12)	0.383

SARS-CoV-2-severe acute respiratory syndrome coronavirus 2, CI-confidence interval, SES-socioeconomic status
